# Maternal feeding strategies and child's food intake: considering weight and demographic influences using structural equation modeling

**DOI:** 10.1186/1479-5868-6-78

**Published:** 2009-11-22

**Authors:** Katja Kröller, Petra Warschburger

**Affiliations:** 1 University of Potsdam, Department of Psychology, Karl-Liebknecht-Str. 24/25, Germany

## Abstract

**Background:**

Research concerning child's food intake have considered various influencing factors, for example parental feeding strategies, demographic and weight factors. At this time, however, there are few findings that explore these factors simultaneously. Accordingly, the aim of this study was to test a structural equation model regarding the associations between maternal feeding strategies and child's food intake.

**Methods:**

556 mothers and their children between 1 and 10 years of age participated in this cross-sectional study. Besides socio-demographic and weight data, the mothers were asked about their feeding strategies as well as their child's food intake.

**Results:**

The well-fitting model explained 73% of the variance in the child's consumption of healthy and 34% of unhealthy food. In addition to the effect of the mother's social status and the child's age, a rewarding and modeling feeding behavior significantly influenced the child's food intake.

**Conclusion:**

The results highlight the relevance of maternal feeding behavior on the child's food intake. In terms of preventing eating- or weight-related problems, the findings indicate the usefulness of training parents in explicit modeling behavior and avoiding food as a reward.

## Background

In recent years feeding and weight problems in children have gained much public attention due to the increased risk of physical secondary diseases (such as diabetes, joint disorders, and difficulties in breathing) as well as the psychosocial consequences (such as eating or affective disorders, [1]). Besides the genetic and cultural factors that influence children's food preferences and eating behavior, parents play a pivotal role. During early childhood years parents use feeding strategies as one way to influence their child's eating [2].

Several reviews concerning the effects of feeding strategies [3,4] showed evidence of a relationship between feeding behavior, food intake and weight of the child. Despite these findings numerous inconsistencies regarding the effects of different strategies still exist. Restriction (the control about kind or amount of the child's food intake) is the feeding strategy that has most consistently been related to a higher risk for getting overweight. Prospective and experimental studies show a relation to a heavier weight [5,6], a lower ability to regulate energy intake [7,8] and a higher preference for the restricted food [9,10]. However, findings providing evidence of a lower child's energy intake due to tighter parental restriction also exist [10,11].

The majority of findings from prospective and cross-sectional studies regarding pressuring the child to eat more or to eat certain foods showed that these strategies correlated with lower weight [e.g. [6,12]] and a higher intake of fruits and vegetables of the child [13,14]. However, results indicating a more frequent use of parental pressure on the child were also found to be related to heavier weight [15,16] as well as a higher energy intake [12,17] by the child. With regards to using food as a reward, the current results appear consistent: rewarding the consumption of disliked food with unhealthy food or snacks seems to increase the preference for the unhealthy food and decreases the preference for the food that was initially promoted [14,18].

Unfortunately, studies that investigate indirect strategies such as monitoring the child's eating, modeling healthy eating, and giving the child more control over his or her food are still rare. These strategies were reported to have positive effects such as lowering a child's weight, decreasing his or her intake of unhealthy food and increasing their intake of healthy food [cf. [12,17,19]].

The use of feeding strategies and their impact on children's food intake both depend on demographic and weight factors of the child and the parents. One relevant factor is the educational and economic family background. There is evidence of more frequent control over the child's food intake in households with higher socioeconomic status [19,20]. Other studies, which considered different aspects of controlling strategies simultaneously, found a lower use for a restrictive [21] and pressured feeding [6,22] in this group. Regarding the mother's own weight, there were also inconsistent findings which indicated that mothers who weigh more control their child's food intake less [16,20], or more often [21] than mothers who weigh less. This discrepancy can be attributed to the use of different definitions of feeding. Studies that found a positive association between mother's weight and her control over the child's eating usually used a combined factor of rewarding and controlling behavior, whereas contrary results analyzed these behaviors as separate feeding strategies. In regards to the child's weight, results from cross-sectional analyses suggest that mothers, who use less pressure, but more restrictive strategies tend to have heavier children [6,13].

Healthy eating behavior and food intake of children is a highly relevant topic. Therefore it is essential to understand the relationship between feeding strategies and the child's eating in association with sociodemographic and weight factors. Current research has concentrated mostly on either a particular feeding strategy, such as restricting and pressuring, or on combined factors of different feeding aspects. Furthermore, previous studies have typically focused on special foods---such as fruits or sweets---or on specific details of the relationship between parenting and food intake. Hence, the aim of this research is to explore the association of maternal feeding strategies and the child's food intake considering sociodemographic and weight aspects. We investigated the effects of a wide range of parental indirect and direct behaviors as well as unhealthy and healthy foods. While we hypothesized a positive association between the indirect feeding strategies and the child's consumption of healthy food as well as a negative association with unhealthy food, we assume a reverse relationship for the direct feeding strategies. To explore interdependencies between the different variables, effects of different feeding aspects, as well as other relevant influences, are considered simultaneously using structural equation modeling with latent variables.

## Methods

### Sample and procedure

Mothers were recruited from inpatient-clinics (e.g. specialized in child rehabilitation for respiratory diseases, dermatitis or susceptibility to infections), child care centers and online communities. Mothers of children aged one- to ten, which have no restrictions concerning their food intake, were asked to fill in a set of questionnaires concerning their own feeding strategies as well as their child's eating behavior. They were given the choice between paper and online version of the questionnaire. After excluding incomplete data and participants who did not fulfill the criteria (e.g. age of the child), 556 out of 597 mothers were included in the analysis (304 mothers, who filled in the online questionnaire, and 252, who filled in the paper version). The majority of the participants was of German nationality (95.9%) and lived in a partnership (81.6%). The mean age of the children was 4.73 years, and 53.2% were boys. Table 1 illustrates the demographic aspects and the weight of the participants. Preliminary analyses revealed differences between the child's age and the educational level of mothers who filled in the online questionnaire (55%) and those who filled in the paper version (45%): Mothers who had chosen the online version had younger children (*F*(1, 554) = 39.38, *p *< .01) and a higher educational level (*F*(1, 554) = 32.17, *p *< .01). These variables were part of the included covariates in the tested model. Accordingly, differences regarding the recruitment strategies will be controlled.

**Table 1:** Sample description

children (n = 556)	
**Sex**	259 (47%) female; 297 (53%) male

**Age**	M = 4.73 years, SD = 2.38 (1 - 10 years of age) - 186 (33.5%) under 3 years of age - 183 (32.9%) between 3 and 6 years of age - 187 (33.6%) over 6 years of age

**BMI-SDS** (mother's report)	M = -0.19, SD = 1.34 (- 6.85 - 4.07) - 88 (15.5%) underweight (BMI-SDS < 10^th ^perc.) - 411 (72.5%) normal weight (10^th ^perc. ≤ BMI-SDS ≤ 90^th ^perc.) - 68 (12.0%) overweight or obese (BMI-SDS > 90^th ^perc.)

**mothers (n = 556)**	

**Age**	M = 33.34 years, SD = 5.61 (20 - 50 years of age)

**Per capita income **(per month)	M = 731.67 Euro, SD = 282.51 (200 - 2667 Euro)

**educational level** (years in school)	M = 10.97 school years, SD = 1.39 (6 - 12 years of school)

**BMI** (self report)	BMI = 24.52 kg/m^2^, SD = 6.04 (15.89 - 66.78 kg/m^2^) - 33 (5.8%) underweight (BMI < 18.5 kg/m^2^) - 340 (59.9%) normal weight (18.5 kg/m^2 ^≤ BMI ≤ 25 kg/m^2^) - 195 (34.3%) overweight or obese (BMI > 25 kg/m^2^)

## Measures

### Demographic and weight data

The mothers were asked for demographic aspects regarding age and socioeconomic status. The socioeconomic status was a combined measure of the family's monthly net income and the mother's educational level. Income (including earnings as well as unemployment, housing, child or sickness benefits, pension, or other earnings) was calculated with respect to the number of family members living in the household. The educational level was determined by the number of years the mother had spent in school.

All mothers reported their height and weight as well as those of their children. A subsample (n = 136) recruited from inpatient-clinics was additionally weighted by means of a standard beam scale (accurate to 100 g) and measured with a calibrated stadiometer (accurate to 1 cm). The subjective and objective weight data show acceptable correlations (r = .88 for the children's weight and height; r = .99 for the mother's weight and height). Therefore, the mother reported height and weight measures appear to be reasonably valid. Furthermore, since we examined child's weight as an influencing factor for the maternal feeding, mother's report probably hold more relevance than objective data. Accordingly, the reported weight was used to calculate the subjects' BMI. For better comparability of children's weight data, a standardized BMI (BMI-SDS) by means of age and sex [23] was calculated.

### Parental feeding strategies

Maternal feeding behavior was assessed with an instrument (ISS) including well-studied strategies and newly generated, so far unconsidered parental feeding strategies. This instrument would be conceptualized to measure the specific parental feeding behavior. To integrate various approaches the item pool was generated from existent questionnaires (CFQ, CFSQ, [24,25]), as well as focus interviews with experts and mothers. Even though the existent questionnaires were based on different theories, we used the items, which characterize concrete feeding behavior. Exploratory factor analyses from a pilot-study with 163 mothers of 3 to 6 years-old children [26] produced 6 scales consisting of 21 items showing a good reliability (more information about the instrument can be furnished from the authors): (1) *restriction *as the extent to which mothers control their child's food (6 items, e.g., "If I did not guide or regulate my child's eating, he or she would eat too much junk food.", α = .75), (2) *monitoring *as overseeing their child's eating (3 items, e.g., "How much do you keep track of the sweets that your child eats?", α = .93), (3) *pressure *as urging their children to eat more food (3 items, e.g., "I have to be especially careful to make sure my child eats enough.", α = .84), (4) *rewarding *as the use of food as a reward (4 items, e.g., "How often during the meal do you encourage your child to eat something by using food as reward?", α = .77), (5) *child's control *as allowing the child to have control over his or her food intake (3 items, e.g., "How often during a meal do you allow the child to eat as much as he or she wants?", α = .73), (6) *modeling *as parents intentional acting as a role model (2 items, e.g. "How often do you eat something you would like your child to eat too?", α = .77). All scales assess the frequency of every feeding strategy on a 5-point Likert scale - ("never" - "always") or a greater agreement ("disagree" - "agree") - with higher scores indicating a more frequent use of the specific strategy.

### Child's food intake

The child's eating was assessed using a food frequency questionnaire adopted for mother's report and included several foods which were relevant to the child's healthy and unhealthy food intake [27]. Mothers indicated on a six-point scale ("never" - "several times a day") how often their children eat certain foods. Based on this information, a score for the number of serves per day was calculated and converted to a 0 to 100 scale, with higher values representing a more frequent consumption. Based on nutritional guidelines for children [27] we related the intake of sweets (like chocolate, cookies, cakes, and candies), salty snacks (pretzels, chips, and nuts), soft drinks (sweetened beverages), and fast food (burger, pizza, hot dogs) to the latent variable of unhealthy food. The intake of fruit (fresh or frozen unsweetened fruits), vegetables (fresh or frozen) and whole grain products (bread, pasta, rice, and cereals with whole grain) were related to the latent variable of healthy food. Previous studies with mothers of children aged 3 to 6 showed an acceptable reliability for the report of healthy (α = .62 - .64) and unhealthy (α = .49 - .62) food [unpublished data]. Correlations between the FFQ and a food report over 3 to 4 days show within a pilot study with 30 mothers and their children (r = .24 - .54) were comparable with other results concerning food reports [unpublished data].

### Statistical Analyses

All statistical analyses were performed using SPSS 15.0. Because missing data rates were below 5%, common Expectation Maximization substitution was applied. The association between maternal feeding strategies and the child's food intake was evaluated by structural equation models using the AMOS 7.0 software package. All variables showed values < 2 for skew and < 7 for kurtosis, indicating a near normal distribution of the data [28]. Model fit was determined using widely accepted relative fit indices [29]: the Comparative Fit Index (CFI > .90), the Tucker-Lewis Index (TLI > .90), and the Root Mean Square Error of Approximation (RMSEA < .06) with a 90% confidence interval.

We followed a two-step approach to evaluate the model, in which first, the measurement models are tested and refined, and then the structural model is tested.

### Statement of Ethics

We certify that all applicable institutional and governmental regulations concerning the ethical use of human volunteers were followed during the research.

## Results

### Measurement models

Structural equation modeling confirmed the factor structure of the questionnaire for feeding strategies (ISS) in this sample. Since previous research [24,25] reveals the dependency of the presented feeding strategies, the tested model allowed correlations between the six factors as well as few relevant item correlations (more information about the model can be furnished from the authors). The six-factor structure demonstrate an adequate fit to the data (Χ^2 ^= 423.47, df = 168, CFI = .94, TLI = .93; RMSEA = .052 [.046 - .059]).

**Figure 1:**
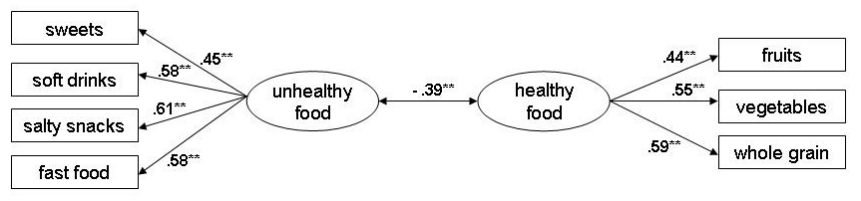
**Model of the factorial structure for child's food intake**.

For the child's food intake we tested a two-factor model for unhealthy and healthy foods (see Figure 1). Due to previous research [30] the parameters were set using a covariance between the healthy and unhealthy food as well as a cross correlation between fruits and vegetables. The tested model shows a good fit: Χ^2 ^= 27.13, df = 13, CFI = .98, TLI = .96, RMSEA = .044 [.020 - .068].

Table 2 summarizes mean and standard deviation of the involved variables.

**Table 2:** Frequency of the maternal use of feeding strategies as well as the child's food consumption (higher values represent more frequent use resp. consumption)

mother's use of feeding strategies			
	**M**	**SD**	**Range**

**modeling**	78.31	15.60	0 - 100
**child's control**	72.08	19.12	0 - 100
**monitoring**	68.51	29.36	0 - 100
**restriction**	46.27	22.94	0 - 100
**pressure**	30.04	30.14	0 - 100
**rewarding**	17.85	17.50	0 - 81.25

**children's food intake**			

	**M**	**SD**	**Range**

**fruits**	78.63	20.52	0 - 100
**vegetables**	69.29	20.31	0 - 100
**whole grain products**	63.34	24.77	0 - 100

**sweets**	51.25	20.79	0 - 100
**chips**	23.94	20.85	0 - 100
**soft drinks**	14.54	22.12	0 - 100
**fast food**	9.87	12.03	0 - 60

### The feeding strategies and child's food intake

**Figure 2:**
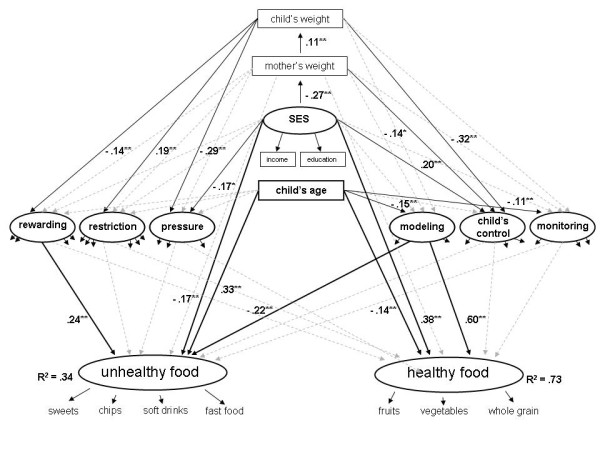
Final model for the association of maternal feeding strategies and the child's food intake considering the impact of covariates (standardized coefficients are given, non-significant coefficients are not included; *p < .05, **p < .01).

Based on the described structure for maternal feeding behavior (6 scales) and children's food intake (2 scales), we tested the structural model of children's consumption of both healthy and unhealthy food affected by maternal feeding strategies. In consideration of the current research [e.g. [6,20,21]] the model integrates the following covariates, which are associated to the parental use of feeding strategies and the child's food intake: the socioeconomic status, child's age and mother's perception on both her and the child's weight. Thus, the model included two foci: first, the influences of the covariates on the use of feeding strategies, and second, the associations between the feeding strategies and the consumption of healthy and unhealthy food beside the effects of covariates. Because of the inconsistent findings regarding the association between feeding strategies and food intake, we allowed all possible paths between these variables. The same applies for the covariates. But to handle the both-way interaction between the child's weight status and the parental feeding strategies, we excluded this path and concentrated on the association between the maternal perception of child's weight and their use of feeding strategies. Because of the widely assumed relation between maternal social status and weight, as well as the mother's and child's weight, we included these associations too. The hypothesized model demonstrates a good fit to the data (Χ^2 ^= 830.91, df = 407, CFI = .92, TLI = .91, RMSEA = .049 [.039 - .048]), see Figure 2. For the purpose of clarity, the following figure only includes the significant regression coefficients for the postulated associations.

Based on this model, maternal feeding strategies, child's age, mother's weight and her socioeconomic status explained 73% of the variance in child's healthy and 34% in child's unhealthy food intake. Regarding the considered first focus - the relation between the demographic and weight variables and the use of feeding strategies - there were several significant effects. Thus, mothers who use more restriction and less rewarding, pressuring and child's control have heavier children. Heavier mothers also allowed their children less control over their food intake. The social status was associated with pressure feeding and child's control: mothers with a higher status gave their children more control and used pressure in feeding more often. Furthermore, there were age related influences, for example mothers with younger children reported a more frequent use of modeling and monitoring. Referring to the second focus - the associations between the involved factors and child's food intake - the rewarding strategy was related to a higher consumption of unhealthy food. Mother's use of modeling was coupled with a strong increase of healthy and a moderate decrease of unhealthy food. In addition, we assumed direct effects of the covariates on the child's food intake too. Whereas the maternal weight status did not show a significant influence, the socioeconomic status and the child's age did. Hence, children with a higher socioeconomic status seemed to consume more healthy food and less unhealthy food. Regarding the influences of child's age, mothers of younger children reported a higher consumption of healthy food and a less frequent consumption of unhealthy food.

## Discussion

The aim of this study was to examine a multi-factorial model of the relationship between maternal feeding strategies and the child's food intake- taking into account the influences of age, socioeconomic and weight factors. Considering the complexity of the child's food intake, our results underline the relevance of feeding strategies (rewarding and modeling) besides the influence of child's age and mother's social status.

As described earlier, former findings on the association between parental feeding strategies and child's food intake have been inconsistent. In order to avoid measurement errors for feeding and food intake as well as the underlying associations of covariates, we analyzed a latent variable model including two foci: the influences of demographic and weight variables on the use of feeding strategies, as well as the effects of various feeding strategies on children's healthy and unhealthy food intake beside the effects of covariates. The hypothesized model showed a good fit for the data. Our results suggest several significant effects which may clarify some of the past inconsistencies. Regarding the first focus - the role of demographic and weight factors - age, weight and socioeconomic status affected the maternal use of feeding strategies. In support with other research [e.g. [5,6,13]], we noticed a more restrictive, but less pressured feeding in heavier children. Furthermore, mothers reported less frequent rewarding and allowed the child less control over his or her food in children with a heavier weight. Our model also indicates significant associations between the social status and the use of parental pressure and allowing the child more control over his or her food intake. This finding was supported by other findings [21,22]. Furthermore, our model shows that mothers use less modeling and monitoring for older than for younger children. Few findings exist regarding the impact of child's age [31,32], indicating a lowered use of direct strategies, like pressuring and restriction, with an older age of the child. Our findings showed a decreased use of indirect strategies suggesting that the older the child, the less the mother uses modeling and monitoring.

Regarding the second focus - the effects on the child's food intake - maternal feeding strategies as well as maternal social background and child's age explained a medium to high amount of the variance. Therefore, the consumption of healthy food was better explained by the included factors (73%) than the consumption of unhealthy food (34%). We can assume that the consumption of unhealthy food is affected by factors other than the consumption of healthy food. There are a few findings that illustrated that with increasing children's age the impact of maternal feeding strategies on the child's eating decreases [31,32]. In terms of the consumption of unhealthy food, the influences of peers and the availability of food seem to be more relevant aspects than parental strategies. However, our findings indicate that a separate examination of healthy and unhealthy food intake could be helpful for future research and that further knowledge is needed to determine factors causing unhealthy eating.

The influence of the mother's socioeconomic status on child's food intake is in line with the widely accepted assumption that families with a lower socioeconomic background report a higher intake of unhealthy food as well as a lower intake of healthy food [30]. There is no significant association between the weight of the mother and the child's food intake, emphasizing that socioeconomic aspects are more important regarding the child's nutrition than the mother's weight. Regarding the child's age a positive correlation was found between age and the consumption of unhealthy food and a negative correlation between age and the healthy food consumption. This is in accordance with food reports done on children of various ages [33] and it underlines the assumption that in the case of older children, mothers use not only less feeding strategies, but also have a decreasing impact on the food intake. This hypothesis needs to be examined further in the future, especially, prospective research on the age related use of feeding strategies and their impact on children's eating patterns.

Besides the influence of child's age and mother's social status there are two strategies that significantly predict the intake of healthy and unhealthy food: rewarding and modeling. Rewarding is associated with an increased intake of unhealthy food, whereas modeling is related to a decrease in unhealthy eating and an increase in healthy eating. Both, the effects of rewarding with food and explicit modeling on the unhealthy eating are in accordance with other findings [13,14,19,34]. For the relationship between rewarding feeding and the consumption of unhealthy food some evidence exists, showing that this feeding behavior increases the preference for the food used as a reward - usually an unhealthy snack [35]. However, on could assume that based on the cross-sectional background of our results that children who eat more sweets and snacks demonstrate a higher responsiveness rate to food as reward, which may be why mothers show a more frequent use of this feeding strategy. The results prove that explicit modeling is even more important for the development of healthy eating habits. A possible explanation for this finding could be the parental role model regarding unhealthy food itself: it could be that it is easier for parents to show an explicit modeling by eating more fruits and vegetables in front of the child than by showing an adequate handling of unhealthy food. Following this theory, most of the parents might eventually prefer to avoid these foods in front of the child, which gives the child no possibility to learn the moderate consumption of these unhealthy foods at all. Considering the higher availability of unhealthy food, along with the increasing influence of peers and the media with age, it is not surprising that parental modeling has a decreased influence on the consumption of unhealthy food.

This study includes the relevant factors that affect the relationship between the parental feeding strategies and the child's food intake in one model. This approach illustrates important associations between the two, but the resulting findings are limited due to its cross-sectional design. Therefore, causal conclusions cannot be drawn for the use of maternal feeding strategies and their impact on child's food intake. Further prospective research is required in order to confirm the effects of age and weight on the child's eating. There are also restrictions regarding the questioned sample. Even though the participated mothers had in generally a higher social background than the representative population of mothers with children those ages, our past findings on a mothers with a lower social status [12] showed comparable results. Additionally, all analyses are based on maternal reports on the use of feeding strategies, their children's food intake as well as height and weight, which may reflect inaccurate self-reporting. We can assume that the maternal perception of these aspects is more relevant than objective data, but these are undeniable highly sensitive questions. Mothers also might have been unwilling to report socially unacceptable behavior and thus may have answered according to social desirability rather than responding truthfully to the questions. It would be useful to integrate more objective measures in future studies.

## Conclusion

Taken together, our results underscore the important influence of parental feeding behavior on the child's eating patterns. This study shows the need for a model that includes healthy and unhealthy food as well as socioeconomic status, age, and weight to detect relevant strategies in this complex association between parental feeding and child's food intake. Taking these results and our past findings on a group with a higher risk for becoming overweight [12] into account, there is some evidence for rewarding as a critical feeding strategy for the child's unhealthy food intake and his or her risk for obesity, whereas modeling seems to have a protective effect. Despite the cross-sectional character of this study, guidelines and trainings regarding the child's eating should focus on these two strategies. Parents should avoid using food as a reward or rewarding the eating of special food. Furthermore, parents should try to act as an explicit model for the consumption of both healthy and unhealthy food. This feeding behavior seems to be one of the most important factors for healthy food intake, but it does show influences on both food aspects. Training programs should focus on guiding parents to be a role model for enjoying unhealthy food in moderation. It would be helpful for parents to learn an adequate consumption of unhealthy food in front of their children, and their influence on regulating this food intake could increase.

## Competing interests

The authors declare that they have no competing interests.

## Authors' contributions

KK conceived of the study, and participated in its design and coordination, performed the statistical analysis and drafted the manuscript. PW steered the design of the study and in the sequence alignment. All authors read and approved the final manuscript.
